# Population structure and associated phenotypes of *Salmonella enterica* serovars Derby and Mbandaka overlap with host range

**DOI:** 10.1186/s12866-016-0628-4

**Published:** 2016-02-04

**Authors:** Matthew R. Hayward, Liljana Petrovska, Vincent A. A. Jansen, Martin J. Woodward

**Affiliations:** Department of Structural and Computational Biology, European Molecular Biology Laboratory, Meyerhofstraße 1, Heidelberg, 69117 Germany; Department of Bacteriology, Animal and Plant Health Agency, Woodham Lane, New Haw, Addlestone, Surrey KT15 3NB UK; School of Biological Sciences, Royal Holloway University of London, Egham, Surrey TW20 0EX UK; Department of Food and Nutritional Sciences, Reading University, Whiteknights, Reading RG6 6AP UK

**Keywords:** *Salmonella enterica*, *S*. Derby, *S*. Mbandaka, Polyphyletic, Host association, SPI-23, IPEC-J2, Biofilm, Pig, Animal feed

## Abstract

**Background:**

The *Salmonella enterica* serovar Derby is frequently isolated from pigs and turkeys whereas serovar Mbandaka is frequently isolated from cattle, chickens and animal feed in the UK. Through comparative genomics, phenomics and mutant construction we previously suggested possible mechanistic reasons why these serovars demonstrate apparently distinct host ranges. Here, we investigate the genetic and phenotypic diversity of these two serovars in the UK. We produce a phylogenetic reconstruction and perform several biochemical assays on isolates of *S*. Derby and *S*. Mbandaka acquired from sites across the UK between the years 2000 and 2010.

**Results:**

We show that UK isolates of *S*. Mbandaka comprise of one clonal lineage which is adapted to proficient utilisation of metabolites found in soya beans under ambient conditions. We also show that this clonal lineage forms a biofilm at 25 °C, suggesting that this serovar maybe well adapted to survival *ex vivo*, growing in animal feed. Conversely, we show that *S*. Derby is made of two distinct lineages, L1 and L2. These lineages differ genotypically and phenotypically, being divided by the presence and absence of SPI-23 and the ability to more proficiently invade porcine jejunum derived cell line IPEC-J2.

**Conclusion:**

The results of this study lend support to the hypothesis that the differences in host ranges of *S*. Derby and *S*. Mbandaka are adaptations to pathogenesis, environmental persistence, as well as utilisation of metabolites abundant in their respective host environments.

**Electronic supplementary material:**

The online version of this article (doi:10.1186/s12866-016-0628-4) contains supplementary material, which is available to authorized users.

## Background

*Salmonella enterica* subspecies *enterica* is an important zoonotic pathogen of warm-blooded vertebrates, with both a broad host species range and geographical distribution. The subspecies can be divided into over 1530 serovars based on the different epitopes of two surface antigens [1]. Epidemiological reports on isolations of *S. enterica* serovars from different livestock species in the UK have revealed interesting trends in association between particular serovars and defined subsets of livestock species [2]. Associations between host species and serovars may reflect the acquisition, by the pathogen, of host adaptations during its evolutionary history [3].

In previous work we characterised two strains of each of *S*. Derby (D1 and D2) and *S*. Mbandaka (M1 and M2) [4–6]. Isolation statistics suggest that these serovars have different host species biases in the UK. *S*. Derby is most frequently isolated from pigs (~50 %) and turkeys (~40 %) and *S*. Mbandaka is most frequently isolated from cattle (~65 %) and chickens (~20 %). During 2013 in the UK, *S*. Mbandaka was the most frequently isolated serovar from animal feed, accounting for 70 of 341 *S. enterica* isolations whereas *S*. Derby accounted for just 4. For the same period there were no isolations of *S*. Mbandaka from pigs and less than 1 % of isolations were made from turkeys, similarly *S*. Derby was not isolated from cattle and less than 1 % of isolations were from chickens [2]. We identified several potential mechanisms pertaining to host adaptation through comparative functional genomics [4]. We demonstrated that *Salmonella* pathogenicity island 23 (SPI-23), an island discovered in the genome of *S.* Derby and absent from the genome of *S*. Mbandaka, plays a role in tissue tropism to porcine jejunum over porcine colon [5]. We also showed that the metabolite utilisation of *S*. Derby D1 and *S*. Mbandaka M1 (found using BIOLOG phenotypic microarray technology) was different at ambient and porcine body temperatures, under aerobic and anaerobic conditions. This data led us to suggest that *S*. Mbandaka may be better adapted than *S*. Derby to using metabolites found in soybean based feeds under ambient conditions [6]. These observations have strengthened our original hypothesis that *S*. Derby isolates D1 and D2 and *S*. Mbandaka isolates M1 and M2 are adapted to distinct niches.

Whilst the observations made on four isolates provide clues on niche and host adaptation, we require a broader population study in order to test the hypothesis that certain genotypes/phenotypes shown by *S*. Derby and *S*. Mbandaka are associated with different biological attributes of the hosts/niches and therefore contribute to the observed isolation bias. Since our original observation of host association was made from the isolation statistics of *Salmonella* incidences in livestock in the UK between 2000 and 2010, we chose to characterise 14 isolates spanning both this location and time. Should there be consistency across this representative population we are better placed to suggest that specific characteristics do contribute to the host distributions. Since the strains used in this study were classified at serogroup level, a typing scheme shown previously to construct paraphyletic groupings, we produced a phylogenetic reconstruction and placed results of genotyping and phenotyping assays in context of this structure [7]. The move from phenotypic to genetic typing also provided the potential to identify if the UK isolations of these serovars reflect one generalist lineage of each serovar or if there were sub-lineages specialised to each host organism.

In the context of the population structure we expand on findings from our previous publications which focused on just two strains of each serovar. We show that the UK isolations of *S*. Mbandaka comprise of one clonal lineage which is adapted to proficient utilisation of metabolites found in soya beans. We also show that this clonal lineage forms a biofilm at 25 °C, suggesting that this serovar may be well adapted to survival *ex vivo*, growing in animal feed. Conversely, we show that *S*. Derby is made of two distinct lineages, L1 and L2. These lineages differ genotypically and phenotypically, being divided by the presence and absence of SPI-23 and a tendency to be more proficient at invading porcine jejunum derived cell line IPEC-J2. We go on to suggest that L1 which is isolated from pigs and turkeys is better adapted to pathogenicity in a porcine host, whereas L2, which has only been recovered from turkeys in the panel of isolates studied here as well as in publicly available MLST profiles, is better adapted to survival *ex vivo*, as it forms a biofilm at 25 °C.

## Results

### Population structure of UK isolates of *S*. Derby and *S*. Mbandaka

**Fig. 1:**
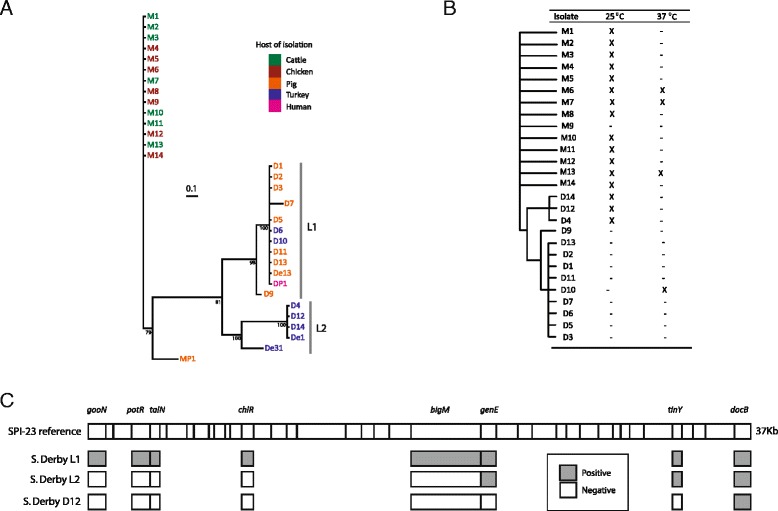
Phenotypic and genotypic differences between *S*. Derby and *S*. Mbandaka isolates. **a** Phylogenetic reconstruction of *S*. Derby and *S*. Mbandaka UK and public ST types. UK isolations of *S*. Mbandaka form one single clonal population with the public isolate MP1 clustering out from the group. Whereas *S*. Derby is formed of two distinct lineages, L1 (UK isolates D3, D5, D6, D7, D9, D10, D11, D1, D2, D13 and public isolates De13 and DP1) and L2 (UK isolates D4, D12, D14 and public isolates De1 and De31). The branch lengths represent the average number of SNPs per partition. Bootstrap values are the mean of the prior probability densities for each node. The first 3000 trees were discarded, the consensus tree was summarised from the remaining 6237 trees, leaving an effective sample size of 451, a stable solution of −5088 and a standard error of the mean of 16.75. Bootstrap values are the mean of the prior probability densities, the branch lengths represent the average number SNPs per partition. **b** Presence and absence of SPI-23 genes and SPI-1 region 1 and 2 in relation to a phylogenetic cladogram. All *S*. Derby L1 isolates lack SPI-1 region 1 and 2, and contain a full version of SPI-23. L2 isolate D12 contains the SPI-23 genes *genE* and *docB* as well as SPI-1 region 1 and 2. *S*. Derby L2 isolates D4 and D14, both contain SPI-1 region 1 and 2 and lack all of SPI-23 with the exception of *docB.* **c** Variation in biofilm formation. All *S*. Mbandaka isolates with the exception of M9 formed a biofilm at 25 °C within 48 h. *S*. Derby L2 isolates formed biofilms at 25 °C, only one L1 isolate, D10, formed a biofilm, this was at 37 °C

The population structure in the UK of *S.* Derby and *S.* Mbandaka was investigated through phylogenetic reconstruction using MLST sequence concatemers of isolates collected between 2000 and 2010. In addition, five public MLST profiles (four *S*. Derby and one *S*. Mbandaka) were added to the analysis to provide wider context to phylogenetic trends. All isolates clustered by their respective serovars (Fig. 1a). Between *S*. Derby and *S*. Mbandaka there were 58 consensus SNPs that distinguished the serovars over the 3336 bps of the MLST sequence concatemer. *S*. Mbandaka isolates formed a single, clonal lineage possessing sequence type ST900, with the exception of the public strain MP1, which possessed MLST type ST206, this particular instance was isolated in 2003 in Canada from a porcine host. *S*. Derby isolates formed two distinct lineages, L1 was isolated from pigs and turkeys (D1, D2, D3, D5, D6, D10, D11 and D13 as well as public isolates DP1 and De13) possessing the sequence type ST40 with the exception of one isolate, D7, which possessed the sequence type ST90. These ST types differ by 11 variant positions spread across all 7 loci. In addition, isolate D9 was typed as ST678; this ST type differed from ST40 at only 5 positions, all in a single loci, *purE*. L2 was formed of isolates made from turkeys (D4, D12, D14 and public isolate De31), with sequence type ST71 as well as public isolate DP1 which possessed MLST type ST72. The two lineages of *S*. Derby were distinguished by 39 consensus SNPs over the 3336 bps of the MLST concatemer. Three public *S*. Derby isolates De1, De13 and De31 were isolated in 1986 in North America from a pig, an unspecified avian species and a turkey respectively. Public *S*. Derby isolate DP1 was isolated in 2003 in Denmark and was of clinical origin. *S*. Derby isolate D8 was MLST typed as ST13 which has been associated with the serovar *S*. Agona, a serovar with a very similar antigenic formula to *S*. Derby [1]. Due to the probable mistyping of this isolate, D8 was removed from all further analysis.

### Distribution of SPI-23 amongst UK isolates of *S*. Derby and *S*. Mbandaka

SPI-23 was present in all *S.* Derby L1 isolates whereas the island was completely missing from L2 isolates D4 and D14 (ST71; Fig. 1c) and all *S*. Mbandaka isolates (ST900). The L2 isolate D12 contained the genes *genE, tinY* and *docB* (Fig. 1c).

### Diversity of SPI-1 amongst UK isolates of *S*. Derby and *S*. Mbandaka

SPI-1 region 1 containing the genes STM2901, STM2902 and STM2903 from *S.* Typhimurium LT2 and region 2 containing the genes SC2837, a putative type III effector protein [4] and SC2838 from *S.* Choleraesuis B67 were absent from all L1 isolates of *S*. Derby but present in all L2 isolates, which contained both regions. All isolates of *S*. Mbandaka contained both regions.

### Diversity in biofilm formation amongst UK isolates of *S*. Derby and *S*. Mbandaka

After 48 h incubated at 25 °C all isolates of *S.* Mbandaka, with the exception of M9, formed a biofilm (Fig. 1b) and three, M7, M6 and M13 formed biofilms at 37 °C also. All L2 *S*. Derby isolates formed biofilms at 25 °C, none of the L1 isolates formed biofilms at 25 °C. L1 isolate D10 was the only isolate of *S*. Derby to form a biofilm at 37 °C.

### Association and invasion of IPEC-J2 monolayers

**Fig. 2:**
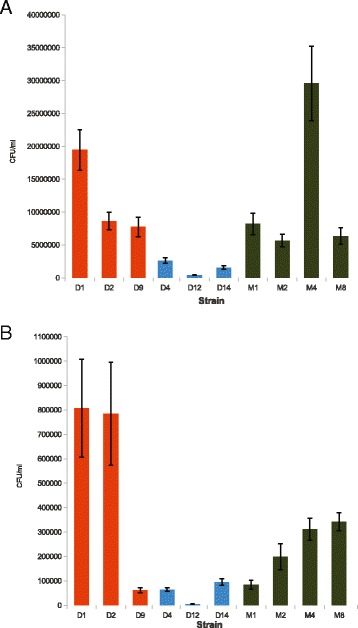
Association and invasion assays. Error bars represent +/-1SEM, bars are coloured based on phylogenetic membership, L1 (orange), L2 (blue) and *S*. Mbandaka (green). **a** Association: There was no lineage or serovar level trend for association to IPEC-J2 assays after 30 min of incubation at 37 °C. *S*. Mbandaka M4 associated in significantly greater numbers to the monolayer then all other isolates (*p* < 0.05). **b** Invasion: *S*. Derby L1 isolates D1 and D2 invaded in significantly greater numbers (*p* < 0.05) then all other isolates. *S*. Derby L2 and L1 isolate D9 invaded significantly fewer numbers than *S*. Mbandaka and lineage 1 isolates

Porcine jejunum derived cell line, IPEC-J2, was used here as a porcine model for studying association to, and invasion of, porcine jejunum by representative isolates of *S*. Derby L1 and L2, and *S*. Mbandaka. *S.* Mbandaka isolate M4 associated to the monolayer in significantly greater numbers (*p* < 0.05) than all isolates with the exception of *S*. Derby D1 (Fig. 2a). *S*. Derby D1 associates in significantly greater numbers (*p* < 0.05) than L2 isolates and *S*. Mbandaka isolates M2 and M8 with the exception of isolate D9. *S*. Derby D4 associated in greater numbers (*p* < 0.05) to the monolayer than other L2 isolate D12.

Unlike the association assays, the proficiency of invasion differed between *S*. Derby L1 and L2 (Fig. 2b). *S*. Derby L1 isolates D1 and D2, invaded the monolayers in significantly greater numbers (*p* < 0.05) than all L2 isolates tested (D4, D12 and D14) with approximately 18 times as many cells internalised, as L1 isolate D9 and *S.* Mbandaka isolates M1 and M2 with more than 5 times the number of internalised cells. *S*. Mbandaka isolates M4 and M8 invaded in significantly greater numbers (*p* < 0.05) than isolates D9, D4, D14, M1 and M2.

### Variability in utilisation of soybean and porcine jejunum and colon homogenates by *S.* Derby and *S.* Mbandaka isolates

**Fig. 3:**
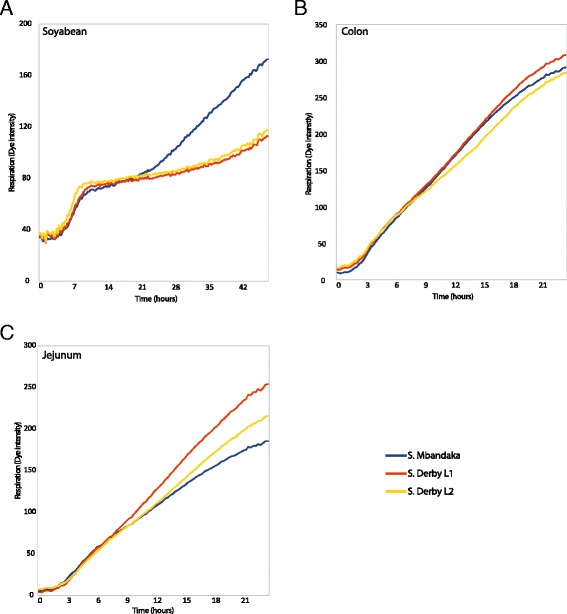
Respiratory dynamics measured through reduction of a tetrazolium dye. All values represent the averaging of 9 replicates of each isolate across a group; L1 (D1, D2, D3, D5, D6, D7, D10, D11 and D13), L2 (D4, D12 and D14) and *S*. Mbandaka. **a** All strains respired on soybean homogenate, though after a period of stationary respiratory dynamics, *S*. Mbandaka isolates underwent a second period of respiration. **b** There was no significant difference between dye intensity values of the three lineages on jejunum homogenate over the 24 h incubation period at 37 °C. **c** The dye intensity for *S*. Derby L1 was significantly different (*p* < 0.05) from that of L2 and *S*. Mbandaka after 13.5 and 10 h respectively, when incubated at 37 °C

For each homogenate, average respiratory rates for the three distinct lineages were produced, L1 (D1, D2, D3, D5, D6, D7, D10, D11 and D13), L2 (D4, D12 and D14) and *S*. Mbandaka. When grown on soybean homogenate *S*. Mbandaka isolates underwent a second period of respiration after the initial plateau phase (Fig. 3a). All *S*. Derby and *S*. Mbandaka isolates respired on porcine jejunum and colon homogenates (Fig. 3b). Absorbance values on jejunum between lineages were not significantly different (*p* > 0.05). Whereas on porcine colon the respiratory rate for *S*. Derby L1 diverged significantly (*p* < 0.05) from L2 and *S*. Mbandaka isolates by 13.5 and 10 h respectively (Fig. 3c).

## Discussion

In this study we performed several genotypic and phenotypic assays on 14 isolates of both *S*. Derby and *S*. Mbandaka isolated between 2000 and 2010 from across the UK. Our data suggest that *S*. Mbandaka isolations are part of a single clonal expansion whereas *S*. Derby is composed of at least 2 sub-lineages. As might be anticipated, the phenotype will reflect the genotype of the lineages and our data from a variety of assays again suggested correspondence to the population structure determined through phylogenetic reconstruction. This was done to assign phenotypic properties to particular genovars of each serovar and we reason that the differential phenotypes may potentially represent adaptations that may in part explain why *S*. Derby and *S*. Mbandaka show different isolation rates from distinct sources.

### Population structure and host range

There is a large diversity in the epitopes of the surface antigens “O” and “H” [1]. Studies have suggested that this diversity is the result of different environmentally derived selection pressures. The “O” antigen is recognised by a diverse range of host specific protozoan predators. As the compliment of protozoan varies amongst different host the selection pressure posed by the predators has driven the diversification of the “O” antigen [8, 9]. The diversity between the two “H” phase antigens has been associated with immunogenicity [10, 11]. It has been proposed that the ability to switch phase allows *Salmonella* to escape detection by the adaptive immune response of the host that has developed in response to the initial phase epitope [12, 13] from the host environment, “O” the gut microflora and “H” the host’s immune system. We may therefore find convergent evolution towards certain epitopes, and therefore certain serogroups, as potential host adaptations. If this is the case, then the evolution of host adaptation amongst *S. enterica* isolates, cannot be studied at the serogroup level of classification alone; we must also have an understanding at the genovar level, to interpret the results of phenotypic studies in relation to host adaptation [7]. The results of phylogenetic reconstructions suggest that *S*. Derby comprises at least two distinct lineages in the UK, with L1 associated with pigs and turkeys and L2 associated with turkeys. It would be interesting to test this hypothesis further by identifying the proportion of porcine and turkey isolates that belong to these two lineages across the UK. It is also evident from the inclusion of public isolates that *S*. Derby L1 and L2 may also be found in North America whereas *S*. Mbandaka in Canada is not from the clonal complex observed in the UK and may include other lineages capable of colonising pigs. The clinical case of *S*. Derby L1 in Denmark opens up the intriguing question of; do the two distinct lineages both have the potential to cause disease in people?

The scale of phenotypic difference between the two lineages would suggest a high degree of genetic difference. We showed that a high number of SNPs had formed between the two *S.* Derby lineages since their divergence. We previously estimated the divergence of *S*. Derby D1 and D2 from *S*. Mbandaka M1 and M2 at between 182kya and 625kya [4]. SNP differences between L1 and L2 account for 67 % of the SNPs between *S*. Derby and *S*. Mbandaka. If we assume a constant substitution rate then we can estimate the split of the two lineages as occurring between 121kya and 419kya. Whole genome alignment, SNP analysis and estimation of the time since divergence between L2 isolate D4 and L1 isolates D1 and D2 is the aim of future work.

*S*. Mbandaka isolates used in this study, and potentially all isolates of UK origin, come from a single lineage with no nucleotide polymorphisms across the seven house-keeping genes. Though notably the public isolate MP1 did not cluster in with this clonal complex. The clonal lineage and the lack of *S*. Mbandaka isolations from pigs in the UK suggests that MP1, isolated from a pig in Canada, may represent as separate lineage with the ability to infect other livestock species [2]. Phenotypic and genotypic diversity amongst UK strains was low and it may be reasonably argued that isolates of this serovar are adapted to both cattle and chickens, or potentially the *ex vivo* environment, with no clear specialist lineage.

### The presence of SPI-23 and not SPI-1 region 1 and 2 correlates with increased invasion of IPEC-J2 monolayers

Interestingly, there was little difference in numbers of cells associating to the porcine monolayer between *S*. Derby L1 isolates and *S*. Mbandaka. From these results it would appear that adhesion to porcine jejunal cells is not an adaptation directly contributing to the high prevalence of *S*. Derby L1 in pigs.

*S*. Derby L1 isolates D1 and D2, possessing MLST type ST40, invaded IPEC-J2 monolayers in significantly greater numbers than isolates from *S*. Derby L2 and *S*. Mbandaka M1 and M2. Conversely, isolate D9 from lineage 1, possessing MLST type ST678, invaded the monolayers in similar numbers to *S*. Derby L2 isolates. This isolate was of porcine origin suggesting that invasion may not be the only adaptation influencing *S*. Derby prevalence amongst pigs. In previous work we have shown the same trend in invasion between *S*. Derby D1 and D2, and *S*. Mbandaka M1 and M2; these strains have been included here as controls, but also to allow direct comparison with a wider population of isolates. *S.* Derby D1 and D2 isolates both contain SPI-23, while *S*. Mbandaka isolates M1 and M2 both lack SPI-23 [4]. The island has been linked with tissue tropism, as it is highly up-regulated in the porcine jejunum and less so in the colon, and contains genes which are essential for adhesion to, and invasion of, IPEC-J2 monolayers [5]. The absence of the same SPI-23 genes in D12 would suggest that they were lost prior to divergence of *S*. Derby L2 and that the remainder of SPI-23 was likely lost from *S*. Derby isolates D4 and D14 recently, over a time period in which no SNPs were formed at the MLST loci. It would also appear that SPI-1 regions 1 and 2 were lost since the divergence of *S*. Derby L1 from L2. The development of a simple PCR for genes between *gooN* and *genE* of SPI-23 could be used as an epidemiological marker for distinguishing between L1 and L2 of *S*. Derby. However, as we have not tested either lineage against a turkey cell line we cannot conclude if the absence of this island signifies a more turkey pathogenic strain but with such a PCR tool assigning lineage with host of isolation would be an interesting avenue to explore in future surveillance.

### Is *S.* Mbandaka adapted towards environmental persistence?

All *S*. Mbandaka isolates with the exception of M9 formed biofilms at 25 °C. *S*. Mbandaka M1 and M2 were both previously shown to contain the gene *sciN*, which is required by *E. coli* to form a biofilm [4, 14]. It is reasonable to suggest that biofilm formation by *S*. Derby L2 isolates at 25 °C appears to have been lost since the split from *S*. Derby L1.

*S*. Mbandaka isolates underwent a second period of respiration on soybean homogenates at 25 °C which did not occur with *S*. Derby isolates. This could reflect catabolite repression, and hence the ability of *S*. Mbandaka to compete with *S*. Derby for the same set of metabolites, until they are exhausted before utilising a second set of metabolites. We showed in previous work that *S*. Mbandaka M1, and not *S*. Derby D1, was able to respire at 25 °C on components of the soybean metabolome: D-saccharic acid, succinic acid, D-trehalose, mellibiose, methyl succinate and fumaric acid [6]. It is possible that *S*. Mbandaka initially uses the same components as *S.* Derby, until they are exhausted, at which point they begin to use other components potentially including the above mentioned metabolites.

Biofilm formation and more proficient use of soybean metabolites, suggest that *S*. Mbandaka is better adapted to survival in the farm environment, potentially persisting in biofilms, using a greater number of soybean metabolites, a common ingredient in animal feed in the UK [15, 16]. This may provide an explanation for the reduced level of antibiotic resistance amongst *S*. Mbandaka strains [2] suggesting persistence in the external farm environment. This could present a model for niche partitioning where one population is better adapted to colonising and persisting in the host and the other adapted to persisting and growing in the external environment: an intriguing hypothesis worthy of further study.

## Conclusion

Further to our previous studies we here extend observations pertaining to potential host adaptations possessed by *S*. Derby and *S*. Mbandaka, made on just 4 strains, to a selection of isolations made from across the UK between 2000 and 2010. The results of phylogenetic reconstruction, based on concatemers of MLST sequences, showed that UK isolates of *S*. Mbandaka were clonal and *S*. Derby isolates were members of at least two lineages. The results of PCR, biofilm, invasion and respiratory assays support our developing hypothesis that S. Derby and *S*. Mbandaka are adapted to separate niches, *in vivo* within a porcine host and *ex vivo* in animal feed, respectively. Further larger-scale studies are required to determine if the results of the current work apply to all isolations of these serovars.

## Methods

### Bacterial strains and culturing

Fourteen strains of each serovar were selected from a collection of over a 1000 agricultural isolates, spanning the period between 2000 and 2010 from different geographical regions across the UK (strain identifiers can be found in [Additional file 1], geographic regions are not shown due to data protection and farmer confidentiality reasons). Strains were stored at−80 °C in 30 % HIB-glycerol throughout the study. Unless stated otherwise, strains were grown at 37 °C for 16 h aerobically on either LB agar plates or in liquid broth vigorously agitated at 220 rpm.

### DNA extraction, MLST and PCR

DNA was extracted using an EasyDNA kit (Invitrogen, UK) as per manufacturer’s instructions. MLST (Multi-Locus Sequence Typing) gene amplification and sequence typing was performed as described on Warwick University MLST webpage [17]. PCR to confirm the presence or absence of SPI-1 regions between STM2901-STM2903 and SC2837-SC2838 and for the SPI-23 genes *gooN, potR, talN, chlR, bigM, shaU, sadZ* and *docB* (primer sequences can be found in [Additional file 2]) were performed using HotStarTaq master mix (Qiagen, USA). PCR cycle conditions were as follows: 15 min at 95 °C, followed by 35 cycles of, 30 s at 95 °C, 30 s at 52 °C and 1 min at 72 °C, followed by 10 min at 72 °C. PCR products were identified through gel electrophoresis and ethidium bromide staining.

### Phylogenetic reconstruction

A phylogenetic reconstruction of the study cohort was produced using the MLST amplicon sequences for UK isolates and all publicly available sequence types for *S*. Derby and *S*. Mbandaka (MLST database accessed on 10/4/15 [17]); which were concatenated and entered into MrBayes v3.1.2 [18] with partitions and unlinked substitution rates for each region. Two independent Metropolis-coupled Markov chain Monte Carlo (MCMCMC) analyses were carried out on a generalised time-reversible model with inverse-gamma substitution rates, consisting each of 3 heated and 1 cold chain, to allow good mixing of parameters. Twenty million iterations, sampling a tree at a 1000 tree interval, were required to achieve a branch split frequency below 0.03. Tracer v1.4 [19] was used to visualise the parameters pertaining to frequency of base substitution and used to identify an adequate burn-in period. SNPs between MLST sequence concatemers were identified using DNAsp [20].

### Association and Invasion of IPEC-J2 monolayers

Association and invasion assays were carried out as previously described using a porcine jejunum derived cell line, IPEC-J2 [21]. In brief, IPEC-J2 (passage 70–72) cells were cultured at 37 °C in aerobic conditions for two days in IPEC-J2 media at which point the monolayers were washed three times with Hank’s Balanced Salt Solution (Sigma, UK). Mid-log cultures of strains D4, D9, D12, M4 and M8 plus previously characterised strains, *S*. Derby D1 and D2 and *S*. Mbandaka M1 and M2 and *Escherichia coli* K12 DH5*α* [5] were produced by diluting overnight cultures 1:100 in LB broth followed by incubation at 37 °C for 3 h with agitation at 220 rpm. Cultures were standardised to an OD_540_ of 1.2 in PBS and diluted 1:20 in IPECs medium. Washed monolayers were inoculated with 1 ml of standardised culture and incubated statically, at 37 °C in 5 % CO_2_ for 30 min. After which they were washed a further three times with Hank’s Balance Salt Solution. The preparations for testing invasion were treated with a 1 % gentamicin (Sigma, UK) solution made up in IPECs medium, and incubated for a further two hours at 37 °C in 5 % CO_2_. After the incubation period, monolayers, of both preparations, were washed a further three times with Hank’s Balance Salt Solution. Monolayers were disrupted with magnetic stirrers in 1 ml of 1 % TritonX (Sigma, UK) made up in PBS. Serial dilutions of each preparation including the initial inoculum were made in PBS from 10^0^–10^-8^ and were plated on to LB agar and incubated at 37 °C for 16 h. Colony forming units (CFUs) were enumerated. ANOVA and Tukey’s HSD tests were performed in R statistical language, where a p-value less than 0.05 was deemed significant [22].

### Respiratory dynamics of isolates grown in homogenates of porcine jejunum and colon, and soybean

Porcine jejunum and colon tissues were collected on two separate occasions. Three, six-week old, cross bred commercial pigs were stunned and euthanized through exsanguination. Jejunum and colon tissues were immediately removed from each pig and stored in ice-cold distilled water. Tissues were cleaned in distilled water to remove luminal content, after which 5 g of tissue was weighed and placed into 10 ml of distilled water. Parallel to this 5 g of soybeans (Natco, UK) were soaked in distilled water for 24 h at room temperature, rinsed, and placed in to 10 ml of distilled water. Tissues and soybeans were homogenised using a D-7801 hand held homogenizer equip with emulsifier blades (Ystral, Germany). The homogenates were diluted 1:10 in distilled water and sterilised by filtration through a 22 nm Stericup filter (Merck Millipore, USA).

Respiratory dynamics were measured in triplicate on three separate occasions. Overnight cultures were standardised to an OD_540_ of 1.2. A 1:5000 dilution of each isolates was made in distilled water. Plates were set up in duplicate for each strain. The wells of a blank BIOLOG PM plate (Biolog, USA), were filled with 100 μl of one of the three homogenates. To each well of the BIOLOG PM plate 1 μl of dye mix A (Biolog, USA) and 1 μl of diluted culture or distilled water (control) was added. Plates were incubated at 37 °C and read every 15 min for 24 h for porcine homogenates and incubated at 25 °C and read every 15 min for 48 h for soybean homogenates using an Omnilog reader (Biolog, USA). T-tests were performed for each time point, where a p-value less than 0.05 was deemed significant.

### Temperature dependent biofilm formation

Crystal violet based biofilm assays were performed in triplicate as described previously [23]. In brief, overnight cultures of all strains with the inclusion of positive biofilm forming control *S*. Enteritidis 27655R and negative non-forming control *S*. Enteritidis 27655S, were grown in LB medium without salt and were standardised to an OD_540_ of 1.2. From this 1 μl was added to a 96 well plate (Iwaki, Japan) containing 200 μl of LB medium without salt. Plates were incubated statically at 25 and 37 °C for 48 h. After which the inoculum was removed and the plates were washed three times with distilled water. Each well was filled with 230 μl of 1 % crystal violet (Pro-lab diagnostics, UK) solution made in water and incubated at room temperature for 30 min. Plates were washed a further 3 times, to remove unbound crystal violet, and dried at 65 °C for 1 h, before 200 μl of acetone was added to each well to lyse cells, suspending the bound crystal violet in solution. Plates were read at 570 nm in a MRX revelation (Dynex Magnellan Biosciences, USA). An OD_570_ above 0.3 was considered to reflect biofilm formation.

### Ethical statement

All studies involving the use of animals were reviewed by the Animal and Plant Health Agency (APHA) ethics committee. However, for the intestinal homogenate studies reported in this manuscript no licenced procedures were performed on live animals, as all animals were euthanized using a schedule 1 approved method prior to post-mortem examination and tissue collection.

### Data availability

All data is provided with the manuscript. MLST sequence types can be downloaded from the *Salmonella enterica* MLST Database [17] using the ST numbers referenced in the text.

## Additional files

Additional file 1:
**Strain information.** Strain identifiers are used throughout the paper for convenience, the isolate number should be used to request culture material. For each strain the year and host of isolation are also displayed. (PDF 20 kb) Additional file 2:
**PCR primer sequences.** PCR primer sequences for the amplification of SPI-1 and SPI-23 gene regions. The product length, start position and the genome from which the primers were designed is also shown. (PDF 28 kb)

## Abbreviations

KYA: thousand years ago
L1: lineage 1
L2: lineage 2
MLST: multi-locus sequence typing
PCR: polymerase chain reaction
SNP: single nucleotide polymorphism
SPI: *salmonella* pathogenicity island
ST: sequence type
UK: United Kingdom
