# Supplementary material for: Population structure and associated phenotypes of Salmonella enterica serovars Derby and Mbandaka overlap with host range
Source: BMC Microbiol. 2016 Feb 4;16:15. doi: 10.1186/s12866-016-0628-4 (PMC4743429; doi:10.1186/s12866-016-0628-4)
Supplement: Additional file 1: — Strain information. Strain identifiers are used throughout the paper for convenience, the isolate number should be used to request culture material. For each strain the year and host of isolation are also displayed. (PDF 20 kb) [file 12866_2016_628_MOESM1_ESM.pdf]

Strain identifier	Serovar	Isolate number	Year	Host
D1	Derby	4052-08	2008	Pig
D2	Derby	4166-08	2008	Pig
D3	Derby	1300-00	2000	Pig
D4	Derby	7725-00	2000	Turkey
D5	Derby	523-02	2002	Pig
D6	Derby	8278-02	2002	Turkey
D7	Derby	1728-04	2004	Pig
D8	Derby	3380-04	2004	Turkey
D9	Derby	6479-06	2006	Pig
D10	Derby	8292-06	2006	Turkey
D11	Derby	1617-08	2008	Pig
D12	Derby	5440-08	2008	Turkey
D13	Derby	L0296-10	2010	Pig
D14	Derby	3315-10	2010	Turkey
M1	Mbandaka	3611-08	2008	Cattle
M2	Mbandaka	5431-09	2009	Cattle
M3	Mbandaka	1045-00	2000	Cattle
M4	Mbandaka	8768-00	2000	Chicken
M5	Mbandaka	3161-02	2002	Chicken
M6	Mbandaka	8766-02	2002	Chicken
M7	Mbandaka	727-04	2004	Cattle
M8	Mbandaka	1223-04	2004	Chicken
M9	Mbandaka	243-06	2006	Chicken
M10	Mbandaka	1789-06	2006	Cattle
M11	Mbandaka	1331-08	2008	Cattle
M12	Mbandaka	4826-08	2008	Chicken
M13	Mbandaka	1015-10	2010	Cattle
M14	Mbandaka	2826-10	2010	Chicken
