# Supplementary material for: Population structure and associated phenotypes of Salmonella enterica serovars Derby and Mbandaka overlap with host range
Source: BMC Microbiol. 2016 Feb 4;16:15. doi: 10.1186/s12866-016-0628-4 (PMC4743429; doi:10.1186/s12866-016-0628-4)
Supplement: Additional file 2: — PCR primer sequences. PCR primer sequences for the amplification of SPI-1 and SPI-23 gene regions. The product length, start position and the genome from which the primers were designed is also shown. (PDF 28 kb) [file 12866_2016_628_MOESM2_ESM.pdf]

Primer Name	Sequence	Amplicon Length	Sequence of origin	Start position
SPI1_G1_F	ctgccagcaggtgaactatt	534	M1	2935546
SPI1_G1_R	aaagcttcatgcaggtcatc			2936080
SPI1_G2_F	tcttcggccatatgattgtt	637	M1	2939573
SPI1_G2_R	acatgcctgtggagttcaat			2940210
gooN_F	atgtcttcccggatataggc	517	D1	2027994
gooN_R	ctggcggatctctttcagta			2028511
potR_F	gacattacggcatcaggaac	684	D1	2032269
potR_R	gcattagctccacagcattt			2031585
talN_F	acgtcagcatccagctttac	224	D1	2033380
talN_R	cgcaatcttcacacactctg			2033156
chlE_F	ccacaaacaaccgaacagat	218	D1	2039475
chlE_R	gaacacatatttcggcatca			2039693
bigM_F	gctgctcacaatcttcctgt	375	D1	2048635
bigM_R	ccgctaataatgggttgatg			2048260
genE_F	cgcagtaaaacaggctcaat	374	D1	2050357
genE_R	tcagatcctgacgtggagtt			2049983
tinY_F	atgcggagctttttaactca	226	D1	2062654
tinY_R	ctctgccagaacggtgtagt			2062428
docB_F	cactccaccgaaagaagaaa	282	D1	2065588
docB_R	attagacgccagcttgtcac			2065870
